# Salt Stress Response of Sulfolobus acidocaldarius Involves Complex Trehalose Metabolism Utilizing a Novel Trehalose-6-Phosphate Synthase (TPS)/Trehalose-6-Phosphate Phosphatase (TPP) Pathway

**DOI:** 10.1128/AEM.01565-20

**Published:** 2020-11-24

**Authors:** Christina Stracke, Benjamin H. Meyer, Anna Hagemann, Eunhye Jo, Areum Lee, Sonja-Verena Albers, Jaeho Cha, Christopher Bräsen, Bettina Siebers

**Affiliations:** a Molecular Enzyme Technology and Biochemistry, Environmental Microbiology and Biotechnology, Centre for Water and Environmental Research, Department of Chemistry, University of Duisburg-Essen, Essen, Germany; b Molecular Biology of Archaea, Institute of Biology II, Albert-Ludwig’s University of Freiburg, Freiburg, Germany; c Institute of Pharmacology and Toxicology, Department of Health, School of Medicine, Witten/Herdecke University, Witten, Germany; d Department of Microbiology, Pusan National University, Busan, Republic of Korea; Kyoto University

**Keywords:** osmoadaptation, *Archaea*, compatible solutes, thermoacidophile, *Sulfolobus acidocaldarius*, TPS/TPP pathway, TreT pathway, trehalose glycosyltransferring synthase, trehalose metabolism, trehalose-6-phosphate phosphatase, trehalose-6-phosphate synthase

## Abstract

The crenarchaeon Sulfolobus acidocaldarius has been described to synthesize trehalose via the maltooligosyltrehalose synthase (TreY) and maltooligosyltrehalose trehalohydrolase (TreZ) pathway, and the trehalose glycosyltransferring synthase (TreT) pathway has been predicted. Deletion mutant analysis of strains with single and double deletions of Δ*treY* and Δ*treT* in S. acidocaldarius revealed that in addition to these two pathways, a third, novel trehalose biosynthesis pathway is operative *in vivo*: the trehalose-6-phosphate (T6P) synthase/T6P phosphatase (TPS/TPP) pathway. In contrast to known TPS proteins, which belong to the GT20 family, the S. acidocaldarius TPS belongs to the GT4 family, establishing a new function within this group of enzymes. This novel GT4-like TPS was found to be present mainly in the *Sulfolobales*. The Δ*treY* Δ*treT* Δ*tps* triple mutant of S. acidocaldarius, which lacks the ability to synthesize trehalose, showed no altered phenotype under standard conditions or heat stress but was unable to grow under salt stress. Accordingly, in the wild-type strain, a significant increase of intracellular trehalose formation was observed under salt stress. Quantitative real-time PCR showed a salt stress-mediated induction of all three trehalose-synthesizing pathways. This demonstrates that in *Archaea*, trehalose plays an essential role for growth under high-salt conditions.

**IMPORTANCE** The metabolism and function of trehalose as a compatible solute in *Archaea* was not well understood. This combined genetic and enzymatic approach at the interface of microbiology, physiology, and microbial ecology gives important insights into survival under stress, adaptation to extreme environments, and the role of compatible solutes in *Archaea*. Here, we unraveled the complexity of trehalose metabolism, and we present a comprehensive study on trehalose function in stress response in S. acidocaldarius. This sheds light on the general microbiology and the fascinating metabolic repertoire of *Archaea*, involving many novel biocatalysts, such as glycosyltransferases, with great potential in biotechnology.

## Abstract (precis)

The metabolism and function of trehalose as a compatible solute in *Archaea* was not well understood. This combined genetic and enzymatic approach at the interface of microbiology, physiology, and microbial ecology gives important insights into survival under stress, adaptation to extreme environments, and the role of compatible solutes in *Archaea*. Here, we unraveled the complexity of trehalose metabolism, and we present a comprehensive study on trehalose function in stress response in S. acidocaldarius. This sheds light on the general microbiology and the fascinating metabolic repertoire of *Archaea*, involving many novel biocatalysts, such as glycosyltransferases, with great potential in biotechnology.

## INTRODUCTION

Microorganisms, including those thriving under hostile conditions, such as (hyper)thermophiles, thermoacidophiles, and extreme halophiles, require efficient adaptation strategies and need to respond to environmental changes and various stresses. Beside known intrinsic factors for the protection of cellular components, such as the structural and mechanistic adaptation of DNA, proteins, and membranes, extrinsic factors like compatible solutes are also essential for the adaptation and stress response. Compatible solutes are low-molecular-weight compounds that do not interfere with metabolism and can therefore be accumulated in large amounts (1). A variety of different compounds serve as compatible solutes, such as polyols (e.g., mannitol and sorbitol), amino acids and their derivates (e.g., glutamic acid, proline, ectoine, and hydroxyectoine), quaternary ammonium salts (e.g., glycine betaine), and disaccharides (e.g., trehalose, sucrose) (1).

The nonreducing disaccharide trehalose (α-d-glucopyranosyl-1,1-α-d-glucopyranoside) is widely distributed in bacteria and eukaryotes, where it functions in general stress protection against heat shock, osmotic stress, desiccation, and cessation of growth (2). Furthermore, trehalose is used as a reserve carbohydrate in some fungi (spore germination) and insects (flight muscles), and some prokaryotes utilize trehalose as a carbon and energy source (3). The function of trehalose, particularly in eukaryotes (fungi and plants), even extends to signaling and regulation as well as to infection mechanisms in pathogenic fungi and bacteria (2, 4–13). In *Eukarya*, trehalose is mainly synthesized by the trehalose-6-phosphate synthase (TPS)/trehalose-6-phosphate phosphatase (TPP) pathway. Only some fungi and algae were reported to utilize the trehalose phosphorylase (TreP) (for trehalose synthesis pathways, see Fig. S1) (10, 12, 14). Conversely, in *Bacteria*, four pathways in addition to the widespread TPS/TPP route have been described: (i) the trehalose glycosyltransferring synthase (TreT) pathway, (ii) the maltooligosyltrehalose synthase (TreY)/maltooligosyltrehalose trehalohydrolase (TreZ) pathway, (iii) the trehalose synthase (TreS) pathway, and (iv) the TreP pathway (6, 14). In numerous cases, a bacterial species harbors more than one pathway in parallel, which all contribute to trehalose synthesis, as shown by mutational approaches (15–24). This led to the assumption that eukaryotes use a single pathway for trehalose synthesis, whereas bacteria employ multiple pathways (6, 10, 14).

Trehalose was also shown to be present in several *Archaea*, mainly in *Crenarchaeota*, including hyperthermophiles of the genera *Thermoproteus* and *Pyrobaculum* and of the order *Sulfolobales* (25). In the thermoacidophiles *Sulfolobus*, *Acidianus*, and *Metallosphaera*, trehalose has been detected as the sole compatible solute, and these organisms lack other common archaeal protective compounds (25). In addition, trehalose was found in some *Euryarchaeota*, such as *Thermoplasma*, and also in some extremely halophilic *Archaea* in which the derivative sulfotrehalose was also detected (25–27). As in *Bacteria*, several pathways for trehalose synthesis have also been detected in *Archaea*, and they often occur in one organism in parallel (14, 28). Biochemical data have been reported for the TPS/TPP pathway in Thermoplasma acidophilum (29) and in halophiles (27), and an unusual complex forming bifunctional TPSP, a fusion protein containing a TPS and TPP domain, was characterized from Thermoproteus tenax (30). TreT enzymes from Thermococcus litoralis (31) and *T. tenax* (28) and the TreY and TreZ enzymes from *Sulfolobus* and *Metallosphaera* spp. (32–43) have been analyzed in detail. TreS has so far been found only in Picrophilus torridus (42, 43). For trehalose degradation, TreH enzymes have also recently been identified (44–48). However, most of the information available on archaeal trehalose metabolism is based on sequence information and analyses of recombinant proteins. Thus, in contrast to *Eukarya* and *Bacteria*, where numerous mutational analyses have been performed (see above), the general function of trehalose in *Archaea* is so far unknown, and the complexity of trehalose metabolism, including the *in vivo* importance of the different synthesis pathways, is not yet understood.

**FIG 1:**
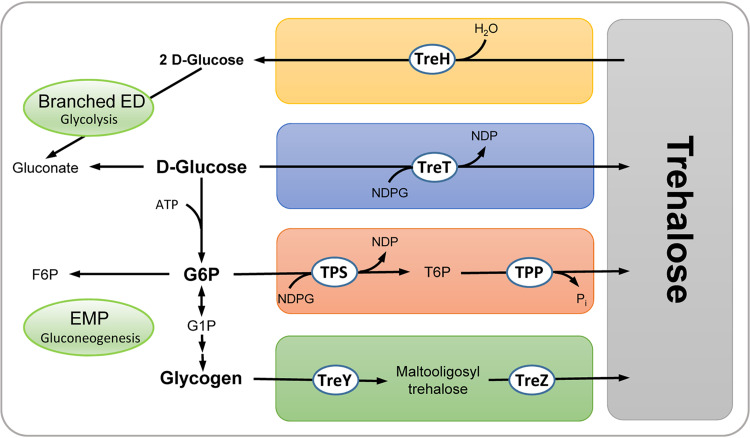
Overview of identified pathways for trehalose synthesis and degradation in S. acidocaldarius. S. acidocaldarius degrades d-glucose via a modified branched Entner-Doudoroff (ED) pathway, whereas the Embden-Meyerhof-Parnas (EMP) pathway is used only for gluconeogenesis (66). Glycogen has been reported as a central carbon storage compound in S. acidocaldarius (61), and hexokinase activity (broad substrate specificity) has been reported for *S. tokodaii* (67). The TreY/TreZ (34, 35), TreT (28), and novel TPS/TPP pathways for trehalose synthesis as well as the trehalases for trehalose degradation (47) are depicted. Abbreviations: TreY, maltooligosyltrehalose synthase; TreZ, maltooligosyltrehalose trehalohydrolase; TPS, trehalose-6-phosphate synthase; TPP, trehalose-6-phosphate phosphatase; TreT, trehalose glycosyltransferring synthase; TreH, trehalase; NDPG, NDP glucose; G6P, glucose 6-phosphate; G1P, glucose 1-phosphate; F6P, fructose 6-phosphate; T6P, trehalose 6-phosphate.

The (hyper)thermoacidophilic crenarchaeon Sulfolobus acidocaldarius, which has optimal growth at pH 2 to 3.5 and temperatures of 75 to 80°C, was shown to accumulate trehalose intracellularly (47). The TreY/TreZ pathway for synthesis has been biochemically characterized (33–35, 39), whereas the TreT pathway was only proposed from sequence data (28) (Fig. 1). Here, we used a combined genetic and biochemical approach and found that both pathways are operative *in vivo* but do not account for trehalose synthesis alone. Instead, a novel, unusual TPS/TPP pathway was identified and characterized and its *in vivo* relevance was confirmed. Furthermore, the accumulation of trehalose by the concerted action of all three synthesis pathways in response to salt stress was demonstrated.

## RESULTS

### Complexity of the trehalose metabolism in S. acidocaldarius—a novel TPS/TPP pathway.

**FIG 2:**
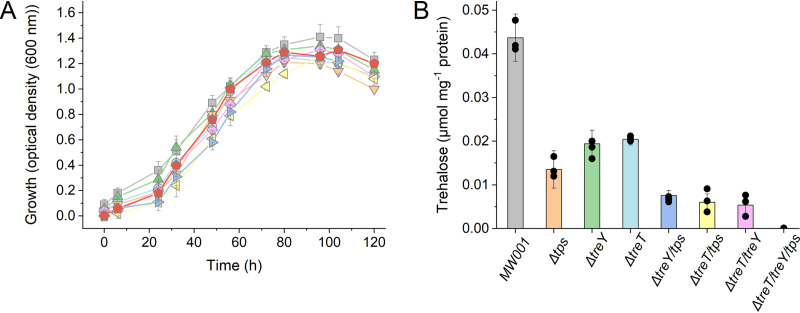
Growth and intracellular trehalose concentrations in S. acidocaldarius MW001 and mutants with deletions of the different trehalose synthesis pathways under standard growth conditions. (A) Growth curves of the parental strain MW001 (gray squares) and mutants with the deletions Δ*treT* (blue circles), Δ*treY* (green triangles), Δ*tps* (orange inverted triangles), Δ*treT* Δ*treY* (pink rectangle), Δ*treT* Δ*tps* (yellow left triangles), Δ*treY* Δ*tps* (purple right triangles), and Δ*treT* Δ*treY* Δ*tps* (red circles) under standard growth conditions. (B) Intracellular trehalose concentrations (in micromoles per milligram of protein) in the parental strain MW001 and of the different deletion mutants in the exponential growth phase. The means and standard deviations for three biological replicates (*n* = 3) are shown.

To elucidate the function of the established TreY/TreZ as well as the predicted TreT pathway in trehalose metabolism of S. acidocaldarius, single- and double-gene-deletion strains (Δ*treY* [*saci*_*1436*], Δ*treT* [*saci*_*1827*], and Δ*treY* Δ*treT*) were constructed. The *treY* gene, which generates the terminal α1-1 linkage, rather than *treZ*, which releases trehalose from glycogen, was deleted to exclude interference with glycogen metabolism. Compared to the reference strain MW001, the Δ*treY* and Δ*treT* single-deletion strains as well as the double-deletion strain (Δ*treY* Δ*treT*) showed no altered phenotype under standard growth conditions (Fig. 2A). The intracellular trehalose concentration of 0.096 μmol mg protein^−1^ in the parental strain was only slightly reduced to 0.040 μmol mg protein^−1^ and 0.032 μmol mg protein^−1^ in the Δ*treY* and Δ*treT* strains, respectively (Fig. 2B). In contrast, the trehalose concentration in the Δ*treY* Δ*treT* double mutant was more significantly reduced, to 0.014 μmol mg protein^−1^ (Fig. 2B). These results showed the significance of both pathways for trehalose synthesis and confirmed that the TreT pathway, which had only been predicted so far, is operative *in vivo*. However, the residual amounts of trehalose in the Δ*treY* Δ*treT* mutant (roughly 10 to 15% that of the reference strain) indicated that S. acidocaldarius harbors an additional, third, yet-unknown trehalose synthesis pathway.

For the identification of the additional trehalose synthesis pathways in S. acidocaldarius, BLAST searches (49) with the P. horikoshii TreT revealed an additional TreT homologue with less similarity and sequence coverage (Saci_1249; E values, 3e^−15^ [P. horikoshii]) than the previously predicted TreT (Saci_1827; E values, 7e^−117^ [P. horikoshii]). The gene (1,335 bp) encodes a protein (444 amino acids) with a calculated molecular mass of 50.3 kDa. The protein is annotated as a GT-B-fold superfamily glycosyltransferase of the GT4 family (www.cazy.org [50]). Homology detection and structure prediction tools (HHPred and Phyre [51, 52]) revealed that Saci_1249 is homologous to both TreT and TPS enzymes.

TPS (Saci_1249) was expressed and purified from Escherichia coli (Fig. S2A). Under denaturing conditions, the enzyme showed a molecular mass of 50 kDa, and under native conditions via gel filtration, it showed a molecular mass of 100 kDa, indicating a homodimeric structure. Notably, despite the similarity to TreT, no TreT activity with glucose and UDPG/ADPG could be determined. Instead, TPS activity in the presence of Mg^2+^ with glucose-6-phosphate (G6P) as the glycosyl acceptor and UDPG or ADPG as the glycosyl donor forming trehalose 6-phosphate was confirmed (Fig. S3). With UDPG as the glycosyl donor, the enzyme showed a *V*_max_ of 6 U mg^−1^ and *K_m_* values of 3.2 mM (G6P) and 3.8 mM (UDPG), corresponding to a *k*_cat_ of 4.9 s^−1^. For ADPG, the *V*_max_ was 4.1 U mg^−1^ and the *K_m_* was 1.8 mM. The S. acidocaldarius TPS was specific for G6P. Glucose 1-phosphate, fructose 1,6-bisphosphate, fructose 1-phosphate, and ribose 5-phosphate could not serve as acceptor substrates (Fig. S3D). The pH optimum of the enzyme was pH 7.0 (Fig. S3E). Also, the thermostability was analyzed; after 2 h at 70°C or 80°C, 68% or 30% residual activity was observed, respectively (Fig. S3F).

For a functional TPS/TPP pathway, a TPP catalyzing the dephosphorylation of trehalose 6-phosphate to trehalose is required. However, no homologues of characterized TPPs were identified in the S. acidocaldarius genome, but 12 members of the HAD superfamily to which canonical TPPs belong were found. Further bioinformatics analyses reduced the number to five promising candidates (Saci_0016, GenBank no. AAY79446; Saci_0094, GenBank no. AAY79521; Saci_0239, GenBank no. AAY79656; Saci_0930, GenBank no. AAY80292; and Saci_1518, GenBank no. AAY80837). We cloned and expressed these genes and confirmed TPP activity, i.e., trehalose formation from trehalose-6-phosphate (T6P), for the purified monomeric Saci_0016 (27 kDa) via thin-layer chromatography (TLC) (Fig. S2B, S4, and S5). In addition, other sugar phosphates, i.e., fructose 6-phosphate, mannose 6-phosphate, and glucose 6-phosphate, were converted, indicating a broader substrate spectrum (Fig. S5A). The activity of the purified enzyme with T6P as the substrate was 8.7 U mg^−1^. With *p*-nitrophenyl phosphate (pNPP) as the artificial phosphatase substrate, the specific phosphatase activity was 0.06 U mg^−1^. Furthermore, the combined trehalose-forming activity from UDPG and G6P via both recombinant enzymes (Saci_1249 and Saci_0016) was observed (Fig. S5B).

To confirm the function of the TPS/TPP pathway in trehalose metabolism of S. acidocaldarius, we constructed the single Δ*tps* mutant (Δ*saci*_*1249*), the double mutants (Δ*treY* Δ*tps* and Δ*treT* Δ*tps*), and the triple mutant (Δ*treY* Δ*treT* Δ*tps*), in which all three assumed pathways for trehalose formation were deleted. As described for the *treY* and *treT* single and double mutants, no altered growth phenotypes under standard conditions for the *tps* single, double, and triple mutants were observed (Fig. 2). The trehalose content compared to that of the parental strain MW001 was decreased in the Δ*tps* single mutant to 0.032 μmol mg protein^−1^. This decrease was even more pronounced in the double mutants (Δ*treY* Δ*tps* and Δ*treT* Δ*tps*) (0.019 to 0.016 μmol mg protein^−1^), and no trehalose was detected in the triple mutant. Hence, all three pathways contribute to the overall trehalose content of the cells. However, since no altered phenotype was observed, trehalose is dispensable under standard conditions, and thus the role of trehalose was not clarified by this set of experiments.

### Physiological role of trehalose in S. acidocaldarius.

**FIG 3:**
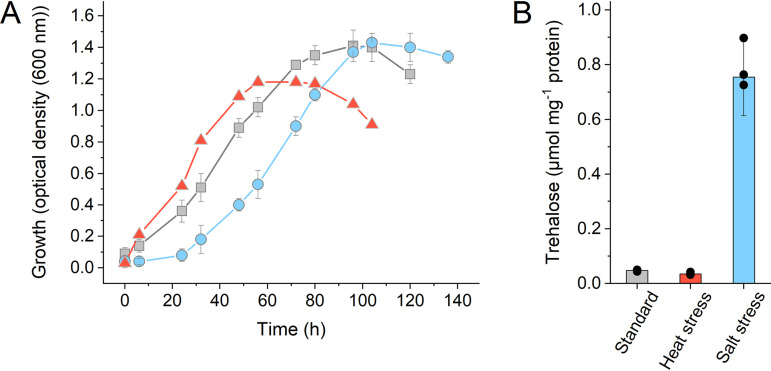
Growth and intracellular trehalose concentration of S. acidocaldarius MW001 under standard growth conditions and under heat and salt stress conditions. (A) Growth curves of the parental strain MW001 under optimal growth conditions (gray squares), under heat stress at 83°C (red triangles), and under salt stress in the presence of 250 mM NaCl (blue circles). Cells were adapted to the respective stress conditions for three passages. (B) Intracellular trehalose concentrations (in micromoles per milligram of protein) in cells from the exponential growth phase grown under standard conditions (gray), under heat stress (red), and under salt stress (blue). The means and standard deviations for three biological replicates (*n* = 3) are shown.

Therefore, we analyzed the impact of the *de novo* trehalose formation under different stress conditions. Under heat stress at 83°C (the optimum growth temperature is 75°C), the growth rate of adapted S. acidocaldarius MW001 cells was not severely affected, but the final optical density at 600 nm (OD_600_) of approximately 1.0 was reduced compared to that at 75°C (OD_600_, 1.4). However, no trehalose accumulation was observed under heat stress in MW001 (0.087 μmol mg protein^−1^ at 83°C compared to 0.097 μmol mg protein^−1^ at 75°C) (Fig. 3).

For cold shock, S. acidocaldarius MW001 was pregrown at 75°C and then transferred to 65°C, resulting in significant growth retardation. However, the cold shock did not enhance the trehalose content of the cells (0.050 μmol mg protein^−1^) (Fig. S6). These results indicate that trehalose is not involved in thermoprotection in S. acidocaldarius.

**FIG 4:**
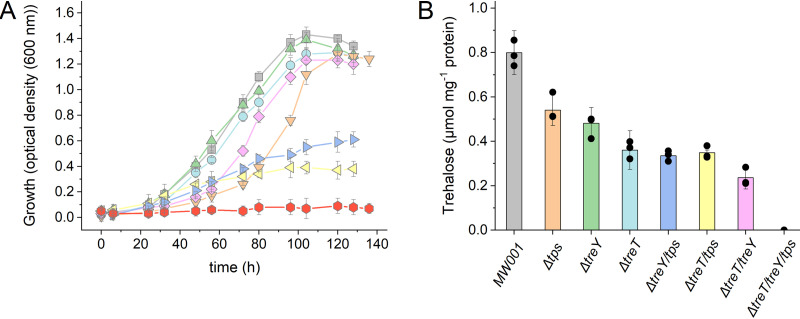
Growth and intracellular trehalose concentration of S. acidocaldarius MW001 and deletion mutants of the different trehalose synthesis pathways under salt stress in the presence of 250 mM sodium chloride. (A) Growth curves of parental strain MW001 (gray squares) and mutants with the deletions Δ*treT* (blue circles), Δ*treY* (green triangles), Δ*tps* (orange inverted triangles), Δ*treT* Δ*treY* (pink diamonds), Δ*treT* Δ*tps* (yellow left triangles), Δ*treY* Δ*tps* (purple right triangles), and Δ*treT* Δ*treY* Δ*tps* (red circles) under salt stress. Cells were adapted to salt stress conditions for three passages. (B) Intracellular trehalose concentrations (in micromoles per milligram of protein) in the parental strain MW001 and the different deletion mutants in the exponential growth phase. The means and standard deviations for three biological replicates (*n* = 3) are shown.

We therefore analyzed the influence of trehalose formation under elevated salinities. All mutant strains constructed were adapted to high salinity (250 mM NaCl, three passages), and the growth phenotype and the intracellular trehalose concentrations were determined in comparison to those of the parental strain, MW001 (Fig. 3 and 4). In the presence of 250 mM NaCl, the reference strain MW001 showed a 12-fold-higher trehalose accumulation (1.12 μmol mg protein^−1^) than under standard growth conditions (0.097 μmol mg protein^−1^) (Fig. 3), although the growth was only slightly delayed. Furthermore, the single-gene-deletion strains (Δ*treY* and Δ*treT*) and the double-deletion strain (Δ*treT* Δ*treY*) showed no altered growth phenotype under salt stress conditions compared to MW001 (Fig. 4A). Conversely, the Δ*tps* single mutant and the double mutants with the *tps* deletion (i.e., Δ*treT* Δ*tps* and Δ*treY* Δ*tps* strains) exhibited a reduced-growth phenotype (Fig. 4A). Growth of the Δ*tps* strain was significantly delayed, although the growth yield was nearly unchanged (Fig. 2A and 4A). However, the Δ*treT* Δ*tps* and Δ*treY* Δ*tps* mutants grew much more slowly, with clearly reduced growth yields (Fig. 4A).

The Δ*treY* Δ*treT* Δ*tps* strain did not contain any measurable trehalose under standard conditions (Fig. 2B). In the presence of 250 mM NaCl, the triple mutant showed no growth and also no intracellular trehalose (Fig. 4). In the single mutants (Δ*treY*, Δ*treT*, and Δ*tps*), the intracellular trehalose concentrations were around 0.499 to 0.714 μmol mg protein^−1^ and thus were reduced by nearly 50% compared to that in the parental strain (Fig. 4B). The double deletions (Δ*treT* Δ*treY*, Δ*treT* Δ*tps*, and Δ*treY* Δ*tps*) resulted in even less trehalose (0.473 to 0.337 μmol mg protein^−1^) (Fig. 4B). These studies highlight the essential role of trehalose in salt stress response in S. acidocaldarius. The complementation of the triple mutant with either of the pathways partially restored growth under salt stress and trehalose formation (Fig. S7). The complementation of the triple mutant with pSVAmz-SH10_tps was expected to result in a growth phenotype resembling that of the Δ*treT* Δ*treY* double mutant, which shows only a slightly reduced growth at high salinities (Fig. 4). The observed growth defect of the complemented strain (Fig. S7) is most likely due to different transcription levels of *tps*, between the native and the constitutively low-expressed promoter of the complementation plasmid. Notably, the addition of trehalose to the medium did not restore the growth of the triple mutant. Also, S. acidocaldarius is not able to utilize trehalose as the sole source of carbon and energy, although a functional trehalase is present, and the addition of trehalose under standard conditions did not result in an altered intracellular trehalose level (data not shown). These results strongly suggest that extracellular trehalose cannot be taken up from the medium and thus that S. acidocaldarius relies on the *de novo* synthesis of trehalose for salt stress response.

**FIG 5:**
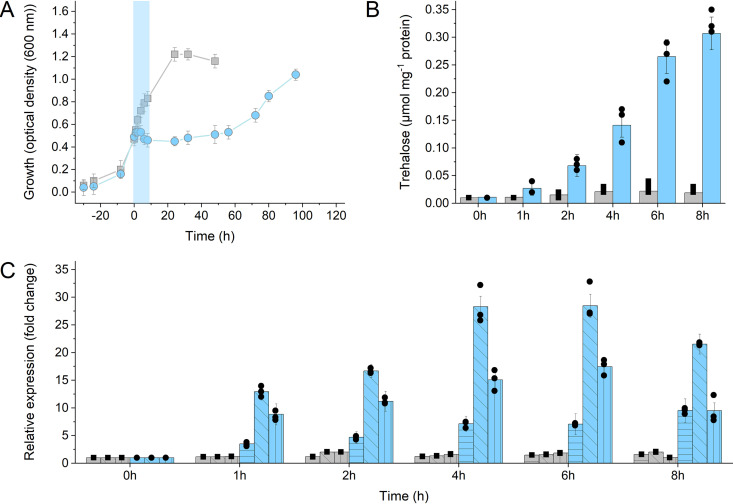
Growth, intracellular trehalose concentrations, and relative expression levels of the trehalose synthesis genes in S. acidocaldarius MW001 under optimal growth conditions and under salt shock conditions. (A) Growth curves of S. acidocaldarius parental strain MW001 under standard growth conditions (gray squares) and under salt shock conditions (blue circles). The cultures were grown under standard growth conditions until exponential growth phase (OD_600_ 0.5–0.6); then, either preheated Brock medium (control) or preheated Brock medium plus NaCl was added (250 mM final concentration). At the times indicated, 10-ml samples were taken for trehalose determination and total RNA isolation for cDNA synthesis. (B) Intracellular trehalose concentration (in micromoles per milligram of protein) in S. acidocaldarius MW001 grown under standard (gray bars) and salt shock (blue bars) conditions. (C) Differential expression of *treY* (horizontally hatched bars), *treT* (diagonally hatched bars), and *tps* (vertically hatched bars) in S. acidocaldarius MW001 under standard conditions (gray bars) and upon salt shock (blue bars). After RNA isolation and cDNA synthesis, qRT-PCR analysis was performed using specific primers for the *treY*, *treT*, and *tps* genes. Relative transcript expression levels of each gene were normalized to that of the internal control gene *secY*. For the growth curves (A) and intracellular trehalose determination (B), the means and standard deviations for three biological replicates (*n* = 3) are shown, and for qPCR (C), means and standard deviations for three technical replicates (*n* = 3) are shown.

To gain further insights into the response of S. acidocaldarius to elevated salinities, the induction of gene expression was analyzed by quantitative reverse transcriptase PCR (qRT-PCR). To this end, the transcription of *treY*, *treT*, and *tps* genes upon NaCl addition to growing cultures of the parental strain MW001 (salt shock) was studied. Samples were taken from the control and salt-induced cultures over a period of 8 h for transcription analysis via qRT-PCR and determination of intracellular trehalose concentration (Fig. 5). After salt stress induction, cell growth was nearly completely arrested and was recovered only after about 60 h of further incubation (Fig. 5A). However, upon salt stress, the trehalose content of the cells continuously increased over the observed 8-h period. As soon as 1 h after induction, a 1.7-fold-higher trehalose concentration was observed in the salt-treated samples, and after 8 h, an 11-fold-higher concentration was observed (Fig. 5B). Accordingly, transcription of all three trehalose genes was induced (Fig. 5C). The qRT-PCR experiments revealed that in salt-stressed cultures, *treT* transcription showed the fastest response (25-fold upregulation after 6 h), followed by *treY* transcription (17-fold upregulated after 6 h), whereas *tps* transcription increased much more slowly but continuously over time (10-fold upregulation after 8 h) (Fig. 5C). This salt-mediated induction of all three trehalose-synthesizing pathways accompanied by trehalose formation confirms the function of trehalose in salt stress response in S. acidocaldarius.

Furthermore, the induction of the trehalose synthesis pathways was confirmed by pathway activities in crude extracts of salt (250 mM NaCl)-stressed cells of S. acidocaldarius MW001. For the TreY/TreZ, TreT, and TPS/TPP pathways, crude extract activities of 3.1 U mg^−1^, 0.071 U mg^−1^, and 0.037 U mg^−1^, respectively, were measured (Fig. S8). In crude extracts of cells grown under standard growth conditions, the respective pathway activities were not detectable using the same assay conditions. In the single-deletion strains (Δ*treT*, Δ*treY*, and Δ*tps*), the corresponding activities were not detected under salt stress, thus confirming the successful deletion of the respective genes.

## DISCUSSION

### A novel GT4-like TPS in S. acidocaldarius.

Unexpectedly, S. acidocaldarius could still synthesize trehalose after deletion of the *treY* and *treT* genes, indicating the presence of a third trehalose synthesis pathway. BLAST searches identified Saci_1249 as a distant TreT homologue in S. acidocaldarius, which, however, also showed structural homology to TPS enzymes. This is in accordance with the CAZy classification of Saci_1249 as a GT-B-folded protein retaining glycosyltransferase activity as well as the homodimeric structure (100 kDa), which applies to both TreTs and TPSs (www.cazy.org [50]). In addition, the alignment of Saci_1249 with TreT and TPS sequences shows—according to the GT-B affiliation—some conservation, especially in amino acid residues involved in donor substrate binding (Fig. S9). However, the characterization of Saci_1249 clearly revealed TPS but no TreT activity. Deletion of the *tps* gene resulted in the loss of TPS activity in crude extracts of salt-stressed cells. The triple deletion Δ*treY* Δ*treT* Δ*tps* abolished trehalose formation, and the resulting mutant was not able to grow under salt stress, clearly demonstrating the function of Saci_1249 as a TPS in trehalose biosynthesis. Notably, like TreT enzymes, Saci_1249 is annotated as a member of the GT4 family and thus seems distinct from classical TPSs, which are classified as GT20 family members (www.cazy.org [50]).

Close homologues of the newly identified TPS (Saci_1249) could be identified only within the TACK superphylum, mainly in the *Sulfolobales*, with only very few candidates scattered in the *Euryarchaeota*. Thus, Saci_1249 represents a novel type of GT4-like TPS with a narrow distribution, mainly in the *Sulfolobales*. To our knowledge, this is the first report of TPS of the GT4 family, which is one of the largest GT families, with ∼150,000 sequences comprising numerous enzyme classes, also including the TreTs (CAZy database).

Moreover, no homologue of canonical TPPs was identified, indicating not only a novel type of TPS in S. acidocaldarius but also a novel TPP. Thus, a completely unusual TPS/TPP pathway is operative in S. acidocaldarius, which is likely restricted to *Sulfolobales*. For the HAD (haloacid dehydrogenase) superfamily member Saci_0016, TPP activity was confirmed, and hence, the enzyme represents a potential TPP candidate. Interestingly, from BLAST searches, it appeared that the distributions of TPS and TPP homologues are roughly similar, and this cooccurrence might support the functional correlation. However, future experiments, including those using gene deletions, are needed to confirm its role in the trehalose metabolism.

### Function of trehalose in S. acidocaldarius.

The presence of trehalose and the biochemistry of some of the synthesis pathways are well established in *Archaea*, and recently, unusual trehalases, presumably for trehalose degradation, were also detected in S. acidocaldarius (for reviews, see references 30 and 47). The intracellular trehalose concentration determined in this work for S. acidocaldarius is well within the range described previously for *Sulfolobales* under standard conditions (25, 47). Here, we show that trehalose is accumulated and essential for growth of S. acidocaldarius under salt stress rather than under temperature stress, to increase the osmolarity of the cytoplasm and thus prevent water efflux from the cells (1).

In other *Archaea*, trehalose accumulation in response to enhanced salinity was also shown for *T. litoralis* (53). However, in this organism, alternative compatible solutes were detected (i.e., mannosylglycerate [MG] and di-myo-inositol-1,1′-phosphate [DIP]), and the significance of trehalose remains unclear. The main compatible solute formed upon salt stress in *Thermococcales* seems to be MG. Furthermore, it is not yet clear whether trehalose is synthesized *de novo* or whether it is taken up from the growth medium (53, 54). Also, in halophilic archaea, trehalose (and sulfotrehalose) was detected with the TPS/TPP pathway as the major synthesis route. However, haloarchaea mainly utilize the “salt-in” strategy and accumulate potassium salts/chloride to counterbalance variable and at least temporarily high environmental salt concentration. In these organisms, trehalose seems to be accumulated in response to decreasing salinities in order to allow survival under low-salt conditions (27). In accordance with the findings for S. acidocaldarius, trehalose accumulation under unfavorable temperature conditions has so far not been reported in archaea. In *T. litoralis* and Pyrococcus furiosus, upon temperature stress, mainly DIP is formed as the key solute (53, 55).

Also in *Bacteria*, if trehalose is used as a compatible solute, it plays a predominant role in salt and osmotic stress adaptation (15–17). However, it was also shown to be involved in heat stress response, for example, in E. coli, Mycobacterium smegmatis (18), and Rhizobium etli (19), as well as to perform additional functions in desiccation tolerance in a few rhizobial species, where it is also involved in symbiosis/nodulation (20–23). Although thermal stress response in S. acidocaldarius was shown to be independent from trehalose accumulation, further functions of trehalose in addition to salinity stress, e.g., in desiccation tolerance or under unfavorable pH or growth conditions, cannot be ruled out and will be addressed in future studies. There are already indications that trehalose is formed in the stationary growth phase (56) and that the TreY/TreZ pathway is upregulated under starvation (57). The recently reported presence of trehalases indicates that the organism is at least able to degrade trehalose (47), and the close relative Saccharolobus solfataricus has already been shown to utilize trehalose as the sole carbon and energy source (58).

### Function of multiple trehalose synthesis pathways.

For many *Euryarchaeaota*, like *Thermococcales* and methanogens, several compatible solutes, such as DIP, MG, and cyclic 2,3-diphosphoglycerate (cDPG), have been described, and trehalose seems to play only a minor or supplementary role, if any. These organisms often harbor only one trehalose synthesis pathway. In contrast, many *Archaea*, which utilize trehalose as a main or exclusive compatible solute, have the tendency to possess multiple (i.e., at least two) synthesis pathways. In general, the presence of multiple synthesis pathways in single organisms is thought to reflect the importance of this compatible solute (12). For *T. tenax*, the unidirectional TreT and the TPSP pathway have been described (28, 30), which from genome analyses is also deduced for other *Thermoproteales*, like *Pyrobaculum* spp. Also, in S. acidocaldarius, trehalose appears to be the exclusive compatible solute. The previously reported functional TreY/TreZ pathway was confirmed (33), and the proposed TreT pathway (encoded by *saci*_*1827*) (28) and the novel TPS/TPP pathway were demonstrated in this study to be operative *in vivo*.

In S. acidocaldarius, all three pathways were found to operate in a concerted manner and contribute to the overall trehalose content of the cells. Notably, complementation of the triple mutant with either one of the pathways restored growth under salt stress and trehalose formation. Interestingly, the Δ*treT* Δ*tps* and Δ*treY* Δ*tps* mutants exhibited a more severely altered growth phenotype than the Δ*treT* Δ*treY* mutant but no difference in the trehalose concentration. This might indicate additional functions of the TPS/TPP pathway enzymes in cellular metabolism, e.g., through substrate promiscuity. Also, a role of the pathway intermediate T6P as a signaling molecule was described especially for fungi and plants (6, 8, 12).

In addition, numerous *Bacteria* employ at least two pathways, TPS/TPP and TreY/TreZ, for trehalose synthesis, although the majority appear to rely solely on the TPS/TPP pathway, which is also the sole biosynthetic route in eukaryotes (1, 14). In *Bacteria*, several mutational approaches have been reported. On the one hand, the results indicate that both pathways contribute to trehalose biosynthesis (15, 20, 22, 59), suggesting a common strategy, with multiple synthesis pathways being responsive to a given stressor like high salinity. On the other hand, e.g., in Corynebacterium glutamicum, the TreY/TreZ pathway predominates under hyperosmotic conditions over the TPS/TPP route (60). Additionally, the TreY/TreZ pathway relies on glycogen/starch as a precursor, which is formed only when sufficient amounts of carbon and energy are available. Thus, the physiological conditions also might trigger the pathway significance, and for C. glutamicum, the interconnection of the TreY/TreZ pathway with the glycogen/starch metabolism has been shown (24). Also, several *Archaea* have been described to utilize glycogen as a storage compound, among them *Sulfolobus* and *Thermoproteus* spp. (61), which also contain the TreY/TreZ pathway. Thus, one might argue that the different pathways starting from different precursors permit the metabolic flexibility to ensure sufficient synthesis of the compatible solute under (rapidly) changing physiological conditions. Future experiments will aim to elucidate the influence of the growth phase and physiological state of the cells as well as of growth substrates on the utilization of the different trehalose synthesis routes.

In conclusion, we unraveled the complexity of trehalose metabolism in S. acidocaldarius. Three pathways are involved in trehalose synthesis, the previously reported TreY/TreZ pathway, the TreT pathway, and a novel TPS/TPP pathway. The latter is characterized by novel enzymes that show no similarity to classical counterparts, comprising a TPS of the GT4 enzyme family as well as a predicted TPP of the HAD enzyme family. Furthermore, we demonstrate that trehalose is formed in response to salt stress in S. acidocaldarius.

## MATERIALS AND METHODS

### Strains and growth conditions.

The E. coli strains were grown in Luria-Bertani medium. S. acidocaldarius MW001 (uracil auxotroph mutant) (62) was grown in Brock’s basal medium at 75°C and pH 3.5 (63) supplemented with 0.2% (wt/vol) N-Z-Amine, 0.1% (wt/vol) dextrin, and 10 μg/ml of uracil. Salt stress experiments were performed in the presence of 250 mM sodium chloride, and temperature stress was tested at 83°C. To this end, cells were adapted for three passages to grow in the presence of elevated salt concentrations or temperature. For the salt shock experiment, cells were grown under standard conditions to an OD_600_ of 0.6 to 0.7 and either prewarmed Brock medium (control) or prewarmed Brock medium with NaCl (250 mM final concentration) was added. For the cold shock experiment, the cells were grown at 75°C (control) and at an OD_600_ of 0.6, the cultures were transferred to 65°C.

### Cloning, heterologous expression, and purification of the recombinant proteins.

The *tps* (*saci*_*1249*, GenBank no. AAY80595.1) and *tpp* (*saci*_*0016*, GenBank no. AAY79446.1) genes were amplified via PCR from genomic DNA of S. acidocaldarius as the template (for primers, see Table 1). PCR fragments were cloned into the vector pET15b (Novagen, USA). Genes were expressed in E. coli BL21(DE3)-CodonPlus induced with 1 mM isopropyl-β-d-thiogalactopyranoside (IPTG). Harvested cells (6,000 × *g*, 20 min, 4°C) were resuspended (1 g cells in 5 ml of 50 mM NaH_2_PO_4_–300 mM NaCl, pH 8) and disrupted with a French press (20.000 lb/in^2^). After centrifugation (4°C, 25,000 × *g*, 45 min), the supernatant was subjected to heat precipitation (75°C, 30 min) and centrifuged. His tagged proteins were purified from the supernatant via Protino Ni-TED (Tris-carboxymethyl ethylene diamine) (Macherey-Nagel, Düren, Germany) following the manufacturers’ instructions. Purified proteins were dialyzed (30 mM Tris–300 mM NaCl, pH 7) and concentrated via ultrafiltration (Vivaspin concentrator; molecular weight [MW] cutoffs, 30 kDa [TPS] and 10 kDa [TPP]; Sartorius, Göttingen, Germany). The proteins were further purified via size exclusion chromatography (Superdex 200 HiLoad 26/60 columns [GE Healthcare Life Sciences, Freiburg, Germany]; buffer, 30 mM Tris–300 mM NaCl, pH 7) and concentrated via ultrafiltration. The pure proteins were used for enzymatic characterization. Purification and molecular mass were monitored by SDS-polyacrylamide gel electrophoresis.

**TABLE 1:** Primers, plasmids, and strains used in this work

Primer, plasmid, or strain	Sequence (5′–3′) or description^*a*^	Source or reference
Primers		
*saci*_1249 *tps* fwd *Nde*I	CCAGGCATATGTTCTCAGTATCTATCAGTA	
*saci*_1249 *tps* rvs XhoI	CGCGCTCGAGCTAAAGGTTAACATTTTTA	
*saci*_0016 *tpp* fwd XhoI	GACGCTCGAGATGAATAAGGACATA	
*saci*_0016 *tpp* rvs BamHI	GCGCGGATCCTTATTTTATAAGAAT	
*saci*_1249 (Δ*tps*) fwd upstream NdeI	GTGAGCATATGCTGTTCTCGTTTACTG	
*saci*_1249 (Δ*tps*) rvs downstream NcoI	GCATCCATGGTGTAATGCGGTAAG	
*saci*_1249 (Δ*tps*) fwd overlapping region	CAATTAGACCTACTAAAGGTTTGAGAACATAAACTAATTTACTCTTTCAG	
*saci*_1249 (Δ*tps*) rvs overlapping region	GTAAATTAGTTTATGTTCTCAAACCTTTAGTAGGTCTAATTGTTATAG	
*saci*_1436 (Δ*treY*) fwd upstream BamHI	GAGGGATCCAGGCTAATAAACTGAACAATG	
*saci*_1436 (Δ*treY*) rvs downstream NcoI	AGCCATGGACTTGCGGAGTTAATAAATG	
*saci*_1436 (Δ*treY*) fwd overlapping region	GGTGGTTGTTGAAATAACGAGGATAGAATTGGTAGC	
*saci*_1436 (Δ*treY*) rvs overlapping region	TTACATTCTAACTAGGGTTGCTGATATCACTGGAACTCTATCC	
*saci*_1827 (Δ*treT*) fwd upstream ApaI	CCCACTGGGCCCCCTCATACCATAAGTGCTAATGGAAC	
*saci*_1827 (Δ*treT*) rvs downstream PstI	CGCCGACTGCAGGCATTAATAGTTTCTGTAGTAGCCTCTGC	
*saci*_1827 (Δ*treT*) fwd overlapping region	CTACCAATTCTATCCTCGTTACTCATATTTCTCTATCATTTCAACAACCACCTCTC	
*saci*_1827 (Δ*treT*) rvs overlapping region	ATGATAGAGAAATATGAGTAACGAGGATAGAATTGGTAGCAGAACTAAGGGTTGG	
*saci*_1827 (*treT*) fwd NdeI	GCACTGGCATATGATAGAGAAATATGAGAAATTTATTG	
*saci*_1827 (*treT*) rvs XhoI	GTCAGACTCGAGTTATACACTATTCCTCTC	
*saci*_1436 (*treY*) fwd NdeI	GTAGAGTTCATATGATATCAGCAACCTACAG	
*saci*_1436 (*treY*) rvs BamHI	GTCACATCGGATCCCTCTATTTTCATATTCTTATTTG	
*saci*_1249 (*tps*) fwd NdeI	CGGCTACATATGTTCTCAGTATCTATCAGT	
*saci*_1249 (*tps*) rvs XhoI	GCTGACTCGAGAAGGTTAACATTTTTACCATAGT	
qRT-PCR primers		
*saci*_0574 (*secY*) fwd	CCTGCAACATCTATCCATAACATACCGA	
*saci*_0574 (*secY*) rvs	CCTCATAGTGTATATGCTTTAGTAGTAG	
*saci*_1827 (*treT*) fwd	GGATATCGGAAGATAAACCGTTAGTAACCC	
*saci*_1827 (*treT*) rvs	AGATCTACGTGCCTCTTAGCTAACTTG	
*saci*_1436 (*treY*) fwd	CAATATGATCAATTCTATAGCCATCAACATC	
*saci*_1436 (*treY*) rvs	ATCCTCCTAGCTATAGACGATTCTTCG	
*saci*_1249 (*tps*) fwd	GCCTCTGATCACCATATTTAACCTGAGG	
*saci*_1249 (*tps*) rvs	CTGTAGGCGGAGTACCAAAGATGATG	

Plasmids		
pET15b	E. coli expression plasmid carrying an N-terminal His tag	Novagen, USA
pSVA407	Gene targeting plasmid, pGEM-T Easy backbone, *pyrEF* cassette of S. solfataricus	(1)
pSVA406	Gene targeting plasmid, pGEM-T Easy backbone, *pyrEF* cassette of S. solfataricus	(1)
pSVAmZ-SH10	Expression plasmid with minimal replicon of pRN1 consisting of the region surrounding orf56 and orf904	Sonja V. Albers
pBS-0539	E. coli expression plasmid of *tps* (*saci*_*1249*) cloned into pET15b	This work
pBS-0586	E. coli expression plasmid of *tpp* (*saci*_*0016*) cloned into pET15b	This work
pSVA1332	In-frame deletion of *treT* (*saci*_*1827*) cloned into pSVA407 with ApaI and PstI	This work
pBS-0285	In-frame deletion of *treY* (*saci*_*1436*) cloned into pSVA406 with BamHI and NcoI	This work
pBS-0584	In-frame deletion of *tps* (*saci*_*1249*) cloned into pSVA407 with NdeI and NcoI	This work
pBS-0627	Expression plasmid of *treT* (*saci*_*1827*) cloned into pSVAmZ-SH10 with NdeI and XhoI	This work
pBS-0806	Expression plasmid of *treY* (*saci*_*1436*) cloned into pSVAmZ-SH10 with NdeI and BamHI	This work
pBS-0626	Expression plasmid of *tps* (*saci*_*1249*) cloned into pSVAmZ-SH10 with NdeI and XhoI	This work

Strains		
E. coli DH5α		Hanahan, USA
E. coli BL21(DE3)		Stratagene, USA
E. coli BL21(DE3) CodonPlus pRil		Stratagene, USA
E. coli ER1821		New England Biolabs, Germany
S. acidocaldarius MW001		(62)
S. acidocaldarius MW001 Δ*treY*		This work
S. acidocaldarius MW001 Δ*treT*		This work
S. acidocaldarius MW001 Δ*tps*		This work
S. acidocaldarius MW001 Δ*treY* Δ*treT*		This work
S. acidocaldarius MW001 Δ*treY* Δ*tps*		This work
S. acidocaldarius MW001 Δ*treT* Δ*tps*		This work
S. acidocaldarius MW001 Δ*treY* Δ*treT* Δ*tps*		This work

a Underlining in sequences indicates the respective restriction sites.

### Generation of deletion mutants of S. acidocaldarius.

For in-frame markerless deletion mutations in S. acidocaldarius MW001, plasmids were constructed essentially as described in reference 62. Briefly, the ∼500-bp upstream and downstream regions of each of the genes (*tps* [*saci*_*1249*, GenBank no. AAY80595.1], *treT* [*saci*_*1827*, GenBank no. AAY81133.1], and *treY* [*saci*_*1436*, GenBank no. NC_007181.1, 1225387 to 1227549]) were amplified by PCR, and the respective up- and downstream PCR products were fused via overlap PCR. The resulting products were cloned into pSVA407 or pSVA406 (for plasmids and strains, see Table 1). Correct cloning was confirmed by sequencing. To prevent plasmid degradation in the recipient strain, all plasmids were methylated by transformation into E. coli ER1821 and after reisolation were transformed into S. acidocaldarius MW001. First selection was performed via uracil auxotrophy and second selection via 5-FOA (5-fluoroorotic acid). Successful gene knockouts were confirmed by PCR and sequencing. For the construction of the complementation plasmids, PCR products of *tps*, *treY*, and *treT* were amplified from genomic DNA from S. acidocaldarius and cloned into the pSVAmzSH10 expression vector (for primers and plasmids, see Table 1). The respective methylated plasmids were transformed into the MW001 triple deletion strain (Δ*treY* Δ*treT* Δ*tps*).

### Enzyme assays.

**(i) TreT.** TreT activity was measured discontinuously in 50 mM HEPES-KOH (pH 7) with 10 mM MgCl_2_, as glucose (10 mM)- and ADP glucose (ADPG, 10 mM)- or UDP glucose (UDPG, 10 mM)-dependent formation of ADP or UDP at 70°C. The formation of UDP or ADP was detected via pyruvate kinase (PK) (rabbit muscle; 8 U) (Merck, Darmstadt, Germany) and l-lactate dehydrogenase (l-LDH) (rabbit muscle; 4 U) (Merck, Darmstadt, Germany) at 55°C in 50 mM HEPES-KOH (pH 7.5), 10 mM MgCl_2_, 2 mM phosphoenolpyruvate, 0.5 mM NADH. In the reverse direction, the glucose formation from trehalose (10 mM) and ADP or UDP (10 mM) was assayed discontinuously in 50 mM HEPES-KOH (pH 7) with 10 mM MgCl_2_, at 70°C. Glucose was determined enzymatically using the glucose dehydrogenase (GDH) from *Pseudomonas* sp. (5 U) (Merck, Darmstadt, Germany) at 37°C in 100 mM HEPES-KOH (pH 7) with 5 mM MgCl_2_ and 5 mM NADP^+^ in a total volume of 500 μl. The reduction of NADP^+^ was measured at 340 nm using a Specord UV/visible-light (Vis) spectrometer (Analytic Jena AG, Jena, Germany).

**(ii) TreH.** Glucose formation from trehalose, was determined in McIlvaine buffer (0.2 M Na_2_HPO_4_, titrated with 0.1 M citric acid to pH 7 at 75°C). The resulting d-glucose was quantified by addition of the reagent DNSA (3,5-dinitrosalicylic acid), incubation for 15 min at 100°C, and absorbance measurements at 575 nm with d-glucose as the positive control (64).

**(iii) TPS.** T6P formation was determined continuously at 340 nm as UDP or ADP release from UDPG or ADPG (10 mM), respectively, and glucose 6-phosphate (G6P, 10 mM) at 55°C (500-μl total volume) by coupling the reaction to the NADH oxidation via PK (8 units) and LDH (4 units) in 50 mM HEPES-KOH (pH 7) containing 10 mM MgCl_2_, 2 mM phosphoenolpyruvate, and 0.5 mM NADH.

**(iv) TPP.** T6P phosphatase activity was determined continuously in 50 mM HEPES/KOH pH 7 with 10 mM MgCl_2_ by following the *para*-nitrophenol release from *para*-nitrophenyl phosphate at 340 nm at 55°C using 4 μg of purified protein.

For preparation of cell extracts, S. acidocaldarius cells were grown under standard conditions or in the presence of 250 mM NaCl until mid-exponential phase (OD_600_ = 0.7 to 0.9). Cells were harvested (8.000 × *g*, 10 min, 20°C), resuspended in buffer, and disrupted (Precellys homogenizer; 3 times for 15 s at 6,500 rpm). After centrifugation, the cell extracts were dialyzed overnight at 8°C (cutoff, 10 kDa). Protein concentrations were determined with a modified Bradford method using bovine serum albumin as a standard (Bio-Rad, Feldkirchen, Germany). All three pathway activities were assayed in 50 mM MES (morpholineethanesulfonic acid)-KOH (pH 6.5), 10 mM MgCl_2_ at 70°C by incubating the respective substrates in the presence of crude extract (100 μg). Samples were taken over time, and trehalose formation was determined with a trehalose assay kit (Megazyme, Bray, Ireland).

### Trehalose determination.

Trehalose concentrations (intracellular) were measured using a trehalose assay kit (Megazyme, Bray, Ireland), according to the manufacturer’s instructions. From 400-ml cultures, 50-ml samples were taken over time; cells were harvested (8,000 × *g*, 10 min, 20°C) and disrupted using a Precellys homogenizer (3 times for 15 s each, 6,500 rpm). After acetone precipitation (80% [vol/vol], 2 h at 4°C), the trehalose concentrations of the samples were determined.

### RNA isolation and RT-qPCR.

Total RNA was isolated from growing cells (5-ml samples, under salt stress and standard conditions) using the FastGene RNA Premium kit (Nippon Genetics, Düren, Germany) according to the manufacturer’s instructions with a prolonged DNase I incubation time (30 min). Salt-stressed cells were washed in Brock basal medium before RNA isolation. Absence of DNA in the RNA samples was confirmed by PCR using *secY* (*saci*_*0574*, GenBank no. AAY79966) primers (primers used in qRT-PCR experiments are listed in Table 1). The quality of RNA samples was analyzed via 1% (wt/vol) agarose gels. First-strand cDNA templates were synthesized using the FastGene Scriptase II synthesis kit (Nippon Genetics, Düren, Germany). The synthesis was performed with random hexamer primers and 1 μg of RNA as the template. Quantitative PCR (qPCR) was performed using qPCRBIO SyGreen Mix Lo-ROX (PCR Biosystems, London, UK) and a MIC (magnetic induction cycler) PCR machine (Bio Molecular Systems, Coomera, Australia). *C_q_* values (quantification cycles) were automatically determined after 50 cycles using *secY* (*saci*_*0574*) as the reference gene (65). qPCRs with DNA-free RNA samples were performed as controls. At least three biological replicates and two technical replicates were performed.

## Supplementary Material

Supplemental file 1
