# Supplementary material for: Salt Stress Response of Sulfolobus acidocaldarius Involves Complex Trehalose Metabolism Utilizing a Novel Trehalose-6-Phosphate Synthase (TPS)/Trehalose-6-Phosphate Phosphatase (TPP) Pathway
Source: Appl Environ Microbiol. 2020 Nov 24;86(24):e01565-20. doi: 10.1128/AEM.01565-20 (PMC7688234; doi:10.1128/AEM.01565-20)
Supplement: Supplemental file 1 [file AEM.01565-20-s0001.pdf]

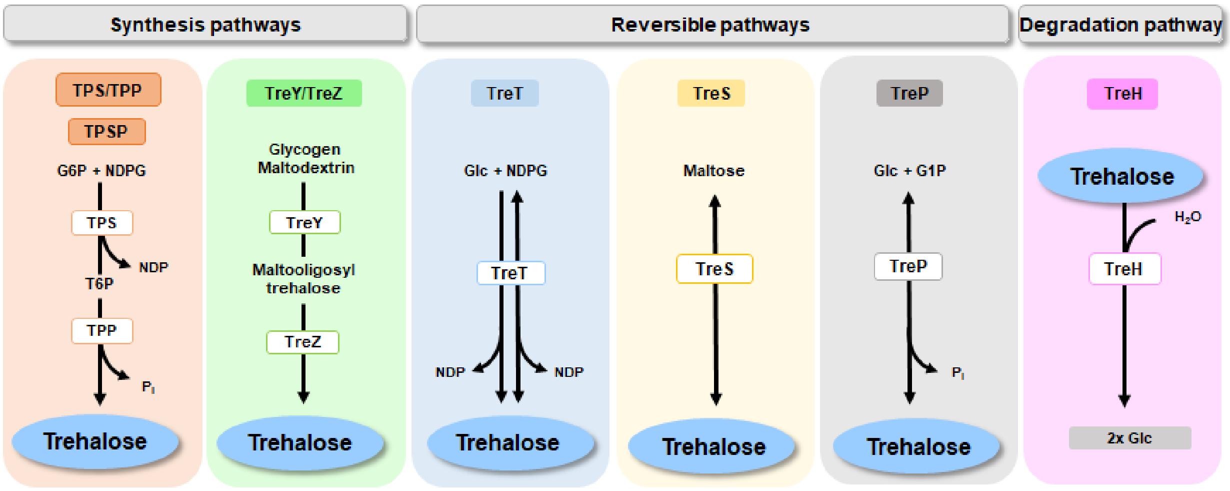

Supplementary figure 1: Trehalose metabolizing pathways including enzymes, substrates and intermediates. Abbreviations; TPS, trehalose-6-phosphate synthase; TPP, trehalose-6-phosphate phosphatase; TPSP, bifunctional fusion protein with trehalose-6-phosphate synthase/trehalose-6-phosphate phosphatase domain; TreY, maltooligosyltrehalose synthase; TreZ, maltooligosyltrehalose trehalohydrolase; TreT, trehalose glycosyltransferring synthase; TreS, trehalose synthase; TreP, trehalose phosphorylase; TreH, trehalase; Glc, glucose; G6P, glucose 6-phosphate; G1P, glucose 1-phosphate, NDP, nucleoside diphosphate; NDPG , nucleoside diphosphate glucose, T6P, trehalose 6-phosphate,

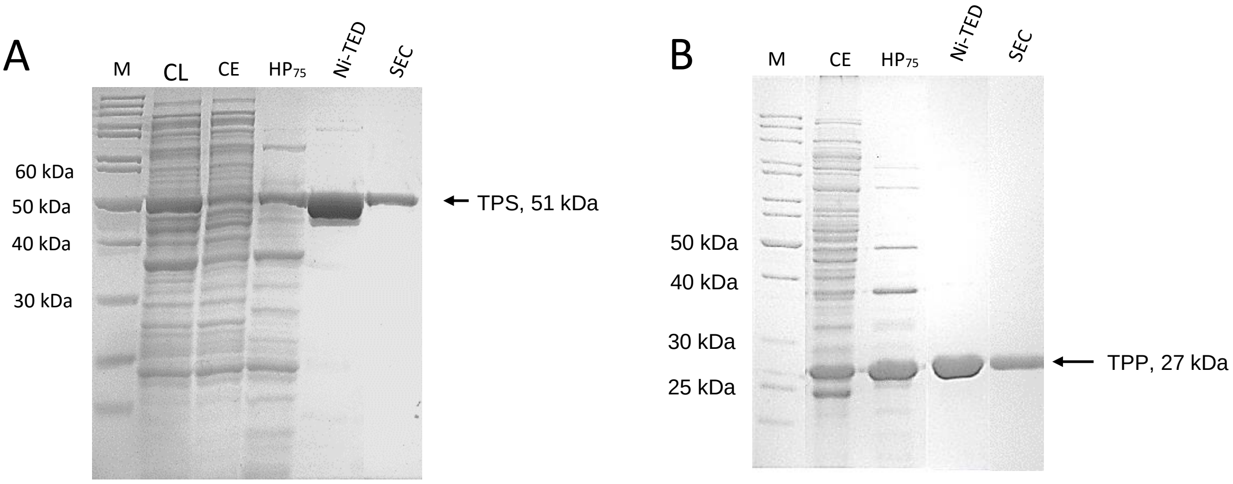

Supplementary figure 2: Purification of the recombinant TPS (A) and TPP (B) from *S. acidocaldarius*. The *tps* gene *saci_1249* and *tpp* gene *saci_0016* were cloned into pET15b and expressed in *E. coli* BL21(DE3)-Codon-Plus with N-terminal His-tag. SDS-PAGE (12.5%, Coomassie staining) of protein fractions (3-10 µg protein) of the respective purification steps are shown. Abbreviations: CL, crude lysate; CE, crude extract; HP_{75}, soluble fraction after heat precipitation at 75°C for 30 min; Ni-TED, protein fraction after Ni-TED affinity chromatography; SEC, protein fraction after size exclusion chromatography; M, protein marker (unstained protein ladder, Thermo Fisher Scientific, Schwerte, Germany).

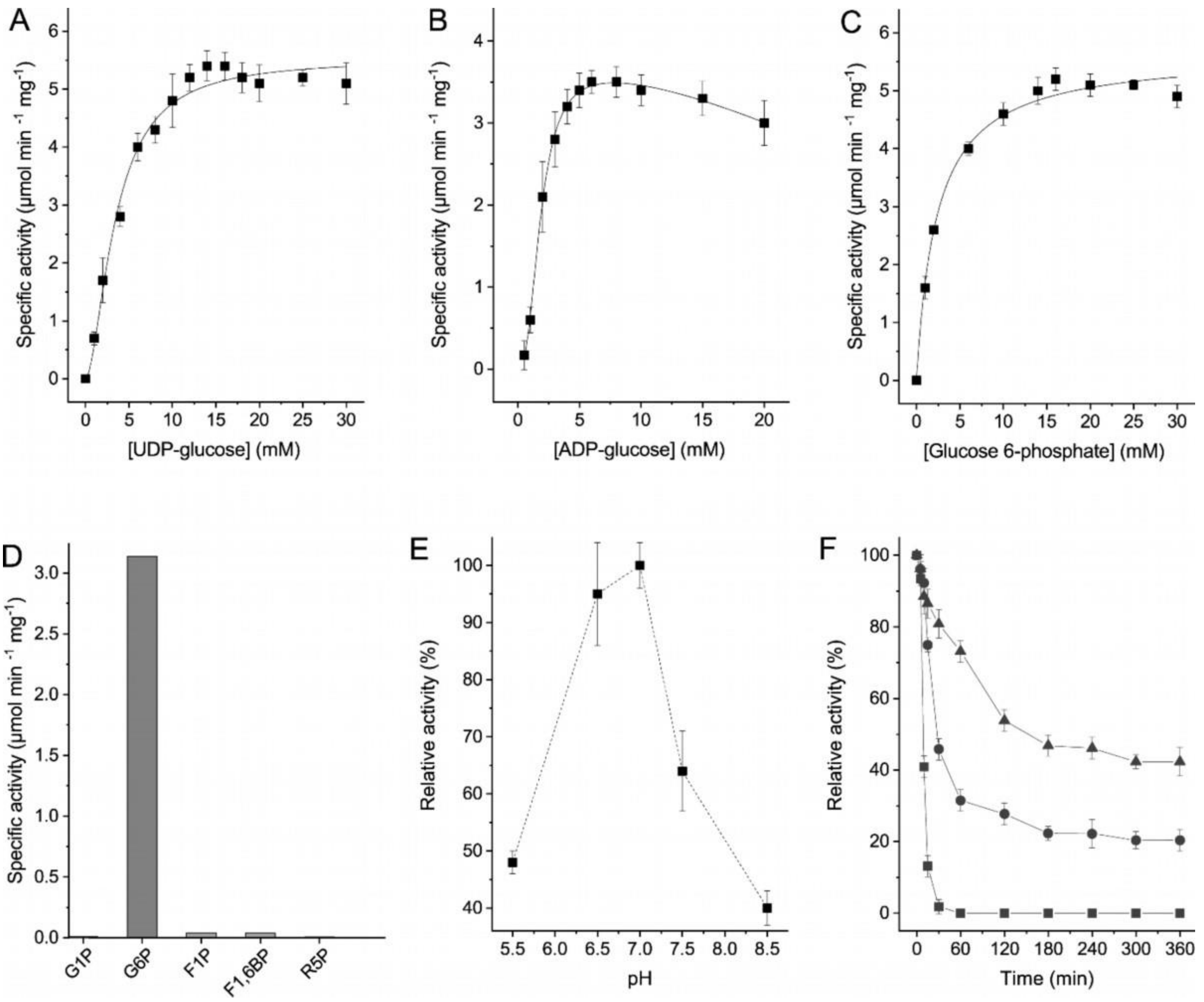

Supplementary figure 3: Characterization of the recombinant TPS from *S. acidocaldarius* in respect to kinetic properties (A-C), substrate specificity (D), pH dependence (E) and thermostability (F). The enzymatic activity **(A-C)** was determined at 55°C (340 nm) using 5 µg of purified enzyme by coupling the formation of UPD or ADP from UDPG or ADPG to the oxidation of NADH with pyruvate kinase (PK) and (lactic acid dehydrogenase (L-LDH) from rabbit muscle as auxiliary enzymes. The specific activity of purified TPS was determined with G6P concentrations from 0-30 mM and 10 mM UDPG **(A)**, UDPG concentrations from 0 - 30 mM and 10 mM G6P **(B)** or ADPG concentrations from 0-20 mM with 10 mM G6P **(C)**. For determination of the substrate specificity **(D)** UDPG was used as glycosyl donor and 10 mM of each glucose 1-phosphate (G1P), glucose 6-phosphate (G6P), fructose 1-phosphate (F1P), fructose 1,6-bisphosphate (FBP) and ribose 5-phosphate (R5P) were used as acceptor substrate. The pH dependence (**E**) of TPS was analyzed in 50 mM TRIS/HCl, MES/KOH and HEPES/KOH. The activity was determined as described above with G6P and UDPG as substrate. The thermostability (**F**) was analyzed at 90°C (triangles), 80°C (circles) and 70°C (squares) over time. Therefore, purified TPS was incubated in 200 mM potassium phosphate buffer pH 7 at the respective temperatures in a thermocycler. At the time indicated samples were removed and the reaction was stopped by incubation for 10-15 min on ice, followed by determination of the residual activity as described). The maximal activity of TPS 5.1 U/mg was defined as 100% activity. Three independent measurements (n=3) were performed and error bars indicate the standard error of the mean (SEM).

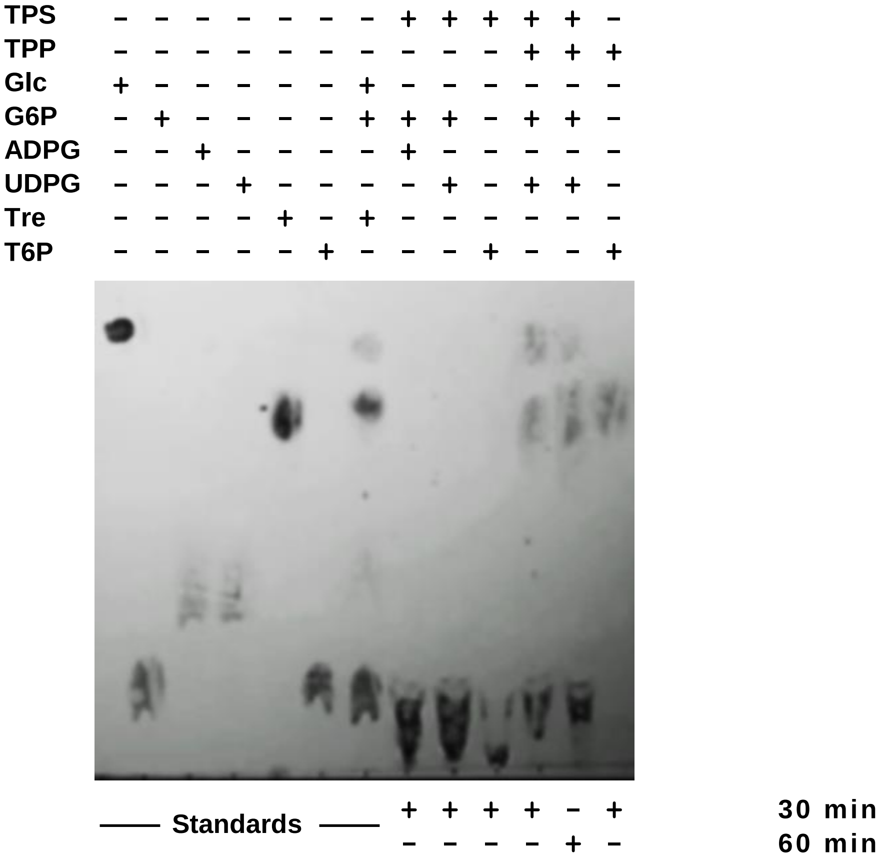

Supplementary figure 4: Analysis of TPS and TPP activity via thin layer chromatography. The TPS and TPP enzyme assays were performed with 10 µg of the purified enzymes in 50 mM HEPES/KOH (pH 7 at 75 °C) with 20 mM $MgCl_2$. The reaction mixtures were supplemented with either 10 mM G6P and 10 mM UDPG or ADPG (TPS) or T6P (TPP reaction) as indicated. After the respective incubation time (30 or 60 min at 55 °C) samples were supplemented with 80% (v/v) acetone and frozen at -20°C. Acetone was removed at 60°C using a Speed Vac concentrator (Eppendorf, Hamburg, Germany). 10 µL of the respective samples as well as standards (3 µl of 10 mM Glc, G6P, ADPG, UDPG, Tre, T6P and a mixture of Glc, G6P and Tre (10 mM each)) were applied and analysed on Kieselguhr coated TLC aluminium sheets (fluorescent indicator F254, 20 x 20 cm, Merck, Darmstadt, Germany) using 1-butanol, ethanol and bidistilled water (5:3:2) as mobile phase. The samples were separated for 5 hours at room temperature and plates were dried and developed with 20 % (v/v) sulphuric acid followed by incubation at 100 °C for 20 min. Abbreviations; TPS, trehalose-6-phosphate synthase; TPP, trehalose-6-phosphate phosphatase; Glc, glucose; G6P, glucose 6-phosphate; ADPG, adenosine diphosphate glucose; UDPG, uridine diphosphate glucose; Tre, trehalose; T6P, trehalose 6-phosphate,

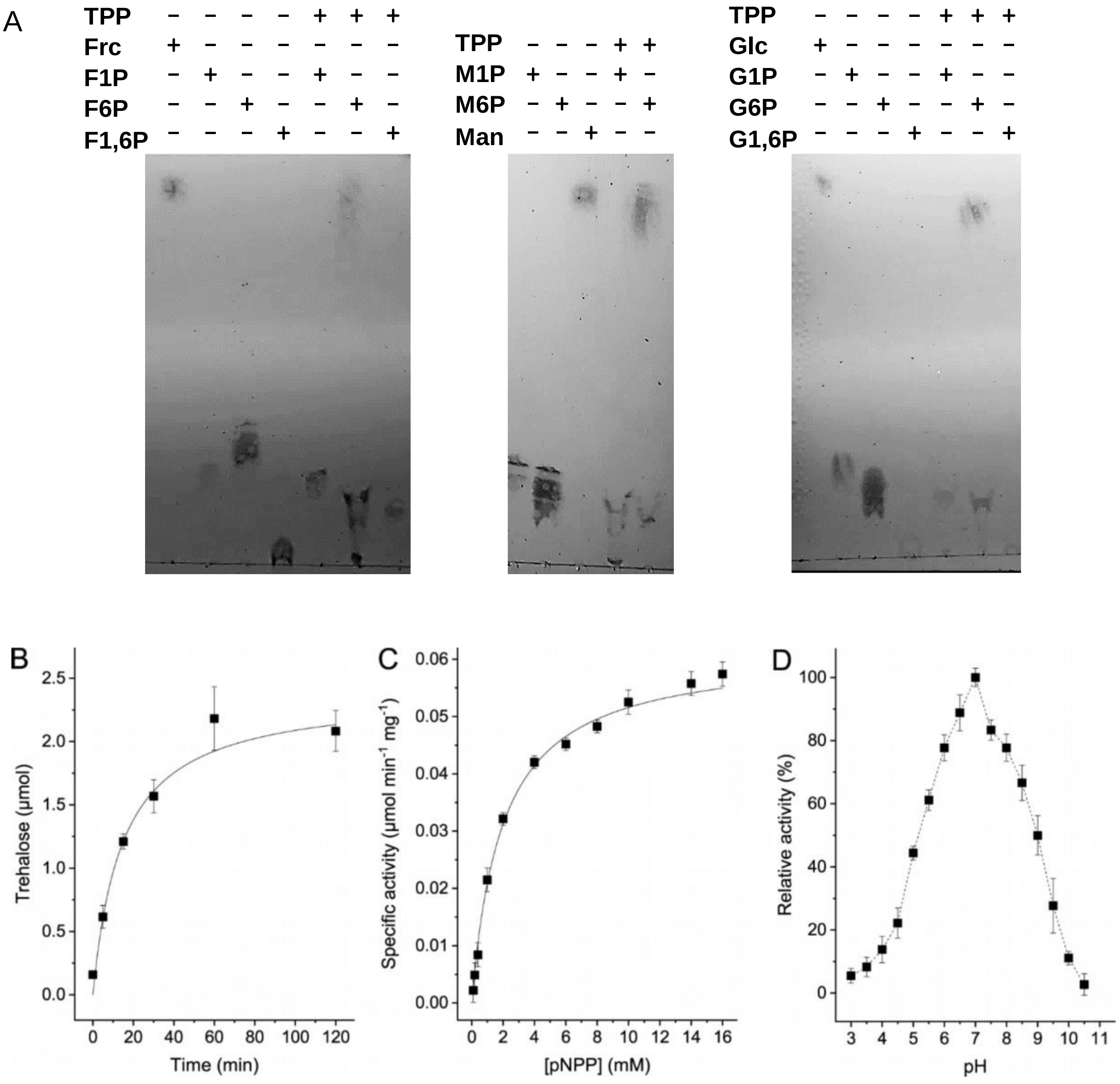

Supplementary figure 5: Characterization of the recombinant TPP from *S. acidocaldarius* in respect to substrate specificity (A), kinetic properties (B, C) and pH dependence (D)*.* TPP substrate specificity for different sugar phosphates was analyzed by TLC **(A).** 3 µl of 10 mM standard solutions of Frc, F1P, F6P, F1,6P, Man, M1P, M6P, Glc, G1P, G6P, G1,6P were applied. TPP reactions were performed with 10 µg of purified enzyme in 50 mM HEPES/KOH (pH 7 at 75 °C) containing 20 mM $MgCl_2$. TLC and plate development was performed as described for supplementary figure 4. TPP activity **(B)** was assayed in a combined assay with the recombinant TPS. Therefore, T6P was first formed by incubation of 10 µg of purified TPS with 10 mM G6P, 10 mM UDPG, 5 mM $MgCl_2$ in 50 mM HEPES/KOH (pH 7.0 at 75°C) for 30 min. Afterwards, 10 µg of purified TPP was added and trehalose formation over time (0 - 120 min) was determined via the Megazyme trehalose assay kit. The initial velocity corresponded to a specific TPP activity of 8.7 U mg^{-1} protein. The general phosphatase activity **(C)** and the pH dependence **(D)** was followed using para-nitrophenylphosphate (pNPP) as substrate. TPP activity was continuously determined at 55°C by monitoring the formation of para-nitrophenol (pNP) from pNPP at 405 nm, using 4 µg of the purified enzyme and substrate concentrations from 0 - 16 mM (0.5 mM for the pH dependence) in 50 mM HEPES/KOH pH 7 with 10 mM $MgCl_2$. For pH dependency the buffers as

described for supplementary figure 3 was used. Three independent measurements (n=3) were performed and error bars indicate the standard error of the mean (SEM). Abbreviations: Frc, fructose, F1P, fructose 1-phosphate; F6P, fructose 6-phosphate; F1,6P, fructose 1,6-bisphosphate; Man, mannose; M1P, mannose 1-phosphate; M6P, mannose 6-phosphate; G1,6P, glucose 1,6-bisphosphate; For additional abbreviations see supplementary figure 4.

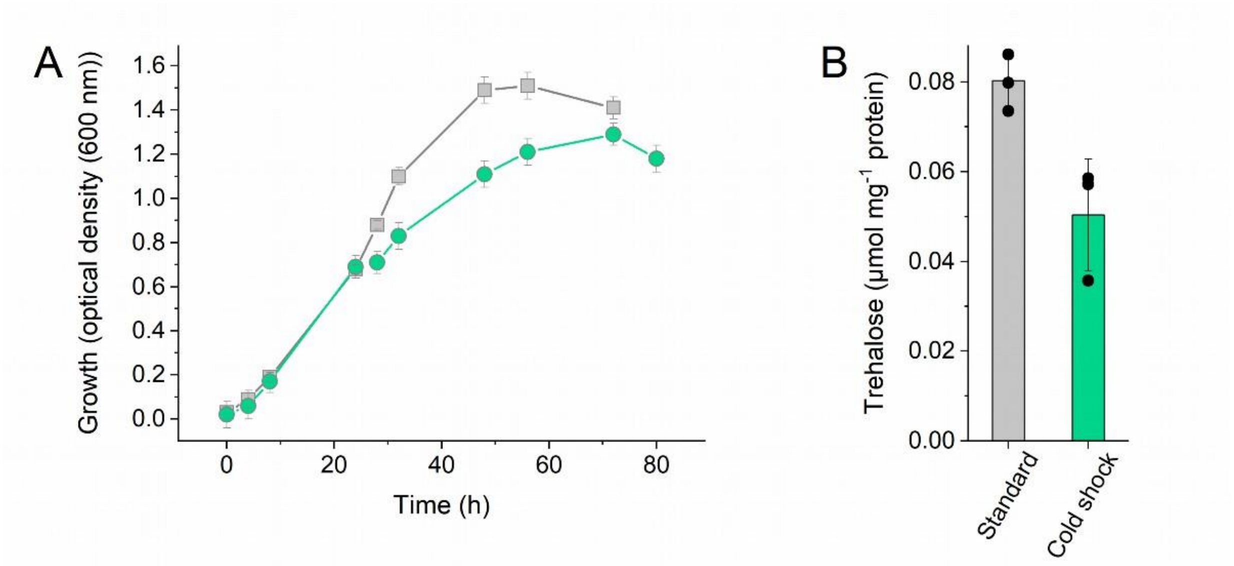

Supplementary figure 6: Growth and intracellular trehalose concentration of *S. acidocaldarius* MW001 under standard growth conditions and under cold shock conditions. Growth curves *S. acidocaldarius* MW001 **(A)** under standard conditions (grey squares) and under cold shock conditions at 65°C (green circles). The cultures were grown under optimal conditions at 75°C until log phase (OD_{600} 0.6), for cold shock conditions the cultures were transferred to 65°C. Intracellular trehalose concentration **(B)** in µmol mg^{-1} protein was determined from cells of late exponential phase (OD 1.0 - 1.1). Three independent measurements (n=3) were performed and error bars indicate the standard error of the mean (SEM).

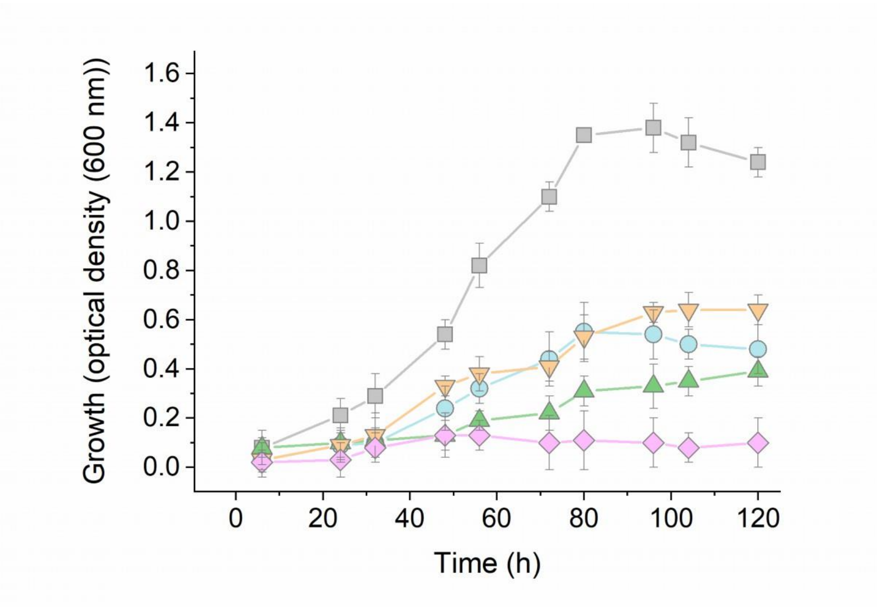

Supplementary figure 7: Growth of the trehalose deficient *S acidocaldarius* MW001 triple mutant strain Δ*treT*/Δ*treY*/Δ*tps* under salt stress conditions complemented with either one of the deleted trehalose synthesis genes. Growth curves of the parental strain MW001 (grey squares) as well as the different (complemented) mutant strains in the presence of 250 mM NaCl is shown. The triple mutant strain Δ*treT*/Δ*treY*/Δ*tps* was transformed with empty plasmid pSVAmz-SH10 as control (pink diamonds), with pSVAmz-SH10_*treT* (blue circles), with pSVAmz-SH10_*treY* (green up triangles), and with pSVAmz-SH10_*tps* (orange down triangles) and was grown on Brock medium supplemented with 250 mM NaCl. Three independent measurements (n=3) were performed and error bars indicate the standard error of the mean (SEM).

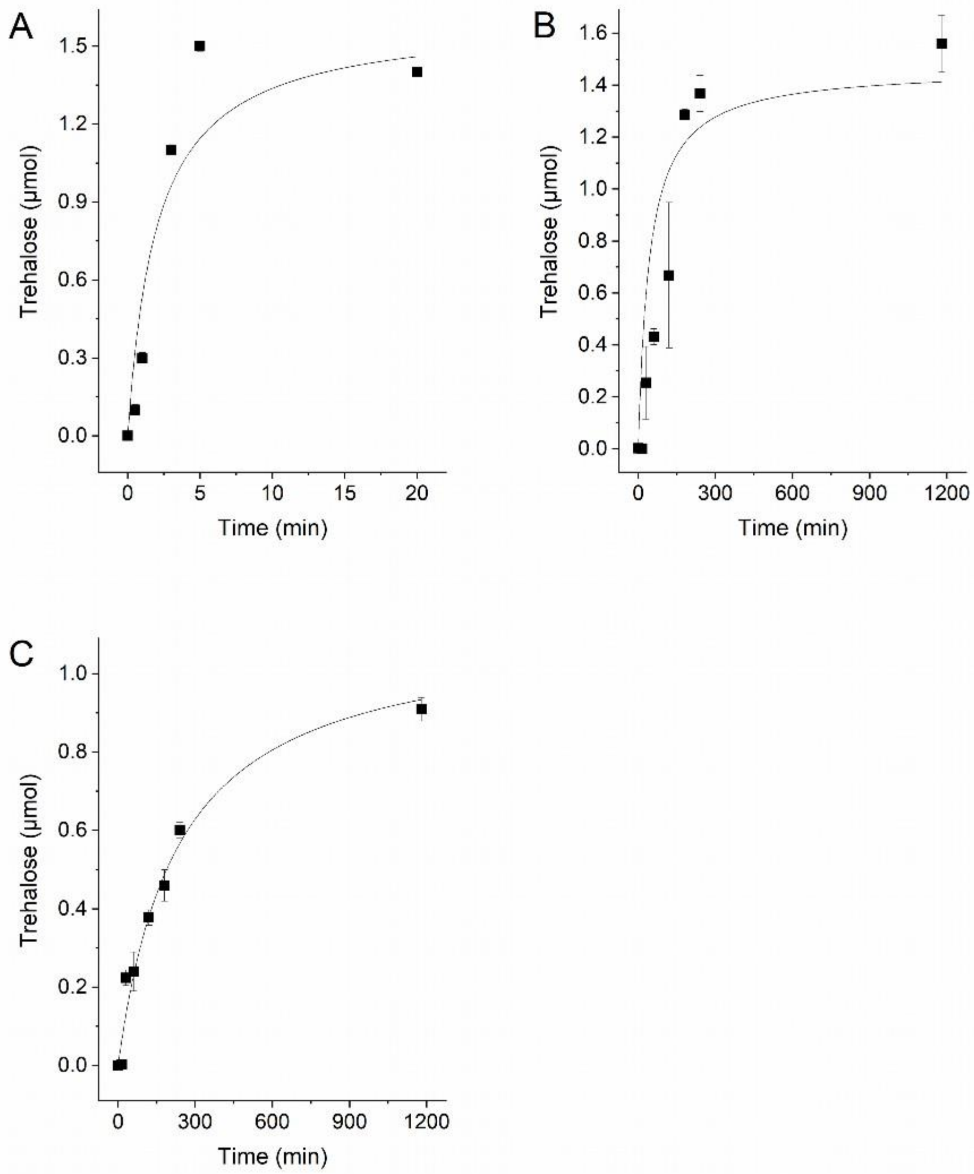

Supplementary figure 8: Activity of the different trehalose synthesizing pathways in crude extracts of salt stressed *S. acidocaldarius* MW001 cells. The enzyme assays were performed with 100 µg of crude extract in 50 mM HEPES/KOH (pH 7.0 at 75°C) supplemented with 5 mM $MgCl_2$. The activity of the TreY/TreZ pathway **(A)** was determined) with 10 mM maltopentaose, of the TreT pathway **(B)** with 10 mM glucose and 10 mM UDPG, and of the TPS/TPP pathway **(C)** with 10 mM G6P and 10 mM UDPG. Samples were taken at the time indicated and trehalose formation was determined via the Megazyme trehalose assay kit. Three independent measurements (n=3) were performed and error bars indicate the standard error of the mean (SEM).

```
TreT_P. horikoshii  26  EVSKIQEKAEKLKGRSFVHV---NSTSFGGGVAEILHSLVPLLRSIGIEARWFVIEGPTEFFN-----V----------- 86
Saci_1249           19  YRDLMDKYGFFELPIELSQLDSSDYYFSVGGVPK----MMLSLINKFNKVRWVSLGPGYPPQV-----KYGDQRLFDFID 89
TPS_E. coli         1   --------MSRLVVVSNRIAPPDEHAASAGGLAV----GILGALK-AAGGLWFGWSGETGNEDQPLKKVKKGNITWASFN 67

TreT_P. horikoshii  87  ------------TKTFHNAL---QGNESLKLTEEMKELYLNVNRENSKFIDLS--SFDYVLVHDPQPAALIEFY---EKK 146
Saci_1249           90  LDPENLKNYTRYKEGIYNES---HGIEKYEIKPSEYISYADYNWISAKKLLEFHNDSDVYFINDFQLLLVGGII---GPS 163
TPS_E. coli         68  LSEQDLDEYYNQFSNAVLWPAFHYRLDLVQFQRPAWDGYLRVNALLADKLLPLLQDDDIIWIHDYHLLPFAHELRKRGVN 147

TreT_P. horikoshii  147 SPWLWRCHIDLSSPN-REFW---EFLRRFVEKYDRYIFHLPEYVQPELDR--------------------NKAVIMPPS 201
Saci_1249           164 APAILWYHIPFVPENLSPRI--RDFIVRSFEGYDYVILSTKRDLEGLLRI-------------------GAKINARQVYPF 223
TPS_E. coli         148 NRIGFFLHIPFPTPEIFNALPTYDTLLEQLCDYDLLGFQTENDRLAFLDCLSNLTRVTTRSAKSHTAWGKAFRTEVYPIG 227

TreT_P. horikoshii  202 IDPLSEKNVELKQT--EILRILERFDVDPEKPIITQVSRFDPWKGIFDVIEIYRKVKEKIPG------VQLLLVGVMAHD 273
Saci_1249           224 IDTSTLRRGSKG----EVDKVKSKYNIGKDEKVITVVARMDPMKSQDVAIMALKKIKES--N------AKLLLVGNGSFT 291
TPS_E. coli         228 IEPKEIAKQAAGPLPPKLAQLKAELK---NVQNIFSVERLDYSKGLPERFLAYEALLEK--YPQHHGKIRYTQIAPTSR- 301

TreT_P. horikoshii  274 D--------P-----EGWIYFEKTLRKIGED-------YDVKVLTNLIGVHAREVNAFQRASDVILQMSIREGFGLTVTE 333
Saci_1249           292 SGALGTNKAG-----NWVRKLQSLASNLGVD-------KKVVFTGHV---SDEELNAIYEASDVIVLPSRIEGFGLVVCE 356
TPS_E. coli         301 --------GDVQAYQDIRHQLENEAGRINGKYGQLGWTPLYYLNQHF---DRKLLMKIFRYSDVGLVTPLRDGMNLVAKE 370

TreT_P. horikoshii  334 AMWKGKP-----VIGRAVGGIKFQIVDGETGFLVR--DANEAVEVVLYLLKHP-EVSKEMGAKAKERVRKNFIITKHMER 405
Saci_1249           357 GWFFEKP-----AIVSSGAGVSELVIDGSNGFVFKSGNYEELAEKIDIVLKEP-DKYSRLSRDTVNKCNVEYAFNQLKDI 430
TPS_E. coli         371 YVAAQDPANPGVLVLSQFAGAANELTS---ALIVNPYDRDEVAAALDRALTMSLAERISRHAEMLDVIV-KNDINHWQEC 446

TreT_P. horikoshii  406 YLDILNSLGG--- 415
Saci_1249           431 FAQAMKDYGKNVN 443
TPS_E. coli         447 FISDLKQIVPRSA 459
```

Supplementary figure 9: Sequence alignment of GT4-like TPS from *S. acidocaldarius* with the TreT from *P. horikoshii* and the classical GT20 TPS from *Escherichia coli*. The alignment was retrieved from HHPred [2] results after analyses with Saci_1249 as template. The amino acid residues shown to participate in acceptor substrate (i.e. G6P) binding in the *E. coli* TPS are highlighted in red those involved in donor substrate (UDPG) binding in blue [3, 4].

References

1. Wagner M, van Wolferen M, Wagner A, Lassak K, Meyer BH, Reimann J, et al. Versatile genetic tool box for the crenarchaeote *Sulfolobus acidocaldarius*. Front Microbiol. 2012;3:214.

2. Zimmermann L, Stephens A, Nam S-Z, Rau D, Kübler J, Lozajic M, et al. A completely reimplemented MPI bioinformatics toolkit with a new HHpred server at its core. J Mol Biol. 2018;430(15):2237-43.

3. Gibson R, P Turkenburg J, Charnock S, Lloyd R, J Davies G. Insights into trehalose synthesis provided by the structure of the retaining glucosyltransferase OtsA. Chem Biol. 2002;9(12):1337-46.

4. Gibson RP, Tarling CA, Roberts S, Withers SG, Davies GJ. The donor subsite of trehalose-6-phosphate synthase: binary complexes with UDP-glucose and UDP-2-deoxy-2-fluoro-glucose at 2 A resolution. J Biol Chem. 2004;279(3):1950-5.
